# Needs and expectations of family caregivers of cancer patients in palliative care: A study protocol

**DOI:** 10.3389/fpsyg.2022.1071711

**Published:** 2023-01-06

**Authors:** Júlio Belo Fernandes, Josefa Domingos, Cidália Castro, Aida Simões, Sónia Fernandes, Ana Silva Almeida, Catarina Bernardes, Luís Miranda, Sandra Risso, Rogério Ferreira, Cristina Lavareda Baixinho, Milene Silvestre, Catarina Godinho

**Affiliations:** ^1^Escola Superior de Saúde Egas Moniz, Almada, Portugal; ^2^Grupo de Patologia Médica, Nutrição e Exercício Clínico (PaMNEC), Almada, Portugal; ^3^Centro de Investigação Interdisciplinar Egas Moniz (CiiEM), Almada, Portugal; ^4^Department of Nursing, Centro Hospitalar de Setúbal E.P.E., Setúbal, Portugal; ^5^Department of Nursing, Centro Hospitalar Barreiro Montijo E.P.E., Barreiro, Portugal; ^6^Departamento de Saúde, Instituto Politécnico de Beja, Escola Superior de Saúde, Beja, Portugal; ^7^Comprehensive Health Research Center, Évora, Portugal; ^8^Nursing School of Lisbon, Lisbon, Portugal; ^9^Nursing Research, Innovation and Development Center of Lisbon (CIDNUR), Lisbon, Portugal

**Keywords:** cancer survivors, palliative care, caregivers, family, need assessment, expectations

## Abstract

Cancer has an associated burden that continues to grow, affecting patients, family caregivers, and the individual’s community. The family caregivers’ unmet needs may harmfully jeopardize their well-being and the patient’s health outcomes. Therefore, it is essential to understand the needs and expectations of family caregivers of cancer patients to develop and improve care practices. The present study aims to explore the needs and expectations of family caregivers of cancer patients in palliative care. This qualitative, descriptive exploratory study will use purposive sampling to recruit family caregivers and healthcare professionals from the palliative care units of two hospital centers in Lisbon and Tagus Valley. First, the Focus group will be performed until data saturation occurs. Then, a conventional thematic analysis will be applied to analyze data with the help of the coding software QDA Miner Lite database. This study’s findings will help identify gaps in care and provide data that can support healthcare professionals in providing evidence-based centered care to family caregivers. It can also generate knowledge that may help stakeholders to develop a comprehensive support system for cancer survivors in palliative care and their family caregivers.

## 1. Introduction

Worldwide, 19.3 million new cancer cases occurred in 2020 (Sung et al., 2021). Cancer is the second leading cause of death globally and accounted for more than 9.6 million deaths in 2018 (World Health Organization, 2022). According to data from the Global Cancer Observatory, in 2020, Portugal registered 60,467 new cases of cancer with 30,168 deaths, being the top five most frequent cancers excluding non-melanoma skin cancer were: colorectum 10,501 (17.4%), breast 7,041 (11.6%), prostate 6,759 (11.2%), lung 5,415 (9%), and stomach 2,950 (4.9%; Global Cancer Observatory, 2021). Leading cancer in terms of risk-attributable deaths for both males and females was lung 4,797 (15.9%), colon 2,972 (9.9%), stomach 2,332 (7.7%), prostate 1,917 (6.4%), and breast 1,864 (6.2%; Global Cancer Observatory, 2021).

This illness has an associated burden that continues to grow, imposing significant physical, emotional, and financial pressure on cancer survivors, and on health and social care systems worldwide (Naughton and Weaver, 2014; Xu et al., 2021; Tran et al., 2022). The Global Burden of Disease collaborators identified significant differences with concern for cancer care and survival. These differences can be linked to lifestyles, risk factors exposure, access to healthcare treatments, and different economic and geographic settings (Tran et al., 2022).

In the specific case of patients with advanced cancer, they should be referred to interdisciplinary palliative care teams. The palliative care teams can offer outpatient and inpatient support for people with advanced cancer, relieving from the symptoms and stress of the illness, as well as active treatment of their cancer (Ghosh et al., 2015; World Health Organization, 2020).

The path to end-of-life can accompany a period of substantial distress for patients and their family caregivers (Dhingra et al., 2018). A family caregiver can be a family member, friend, or another person who has an emotional and social connection with a patient and carries out nonprofessional or unpaid care for patients (Payne, 2010). Family caregivers can provide patients with physical, psychological, social, and existential support. However, while providing this care, they can also experience high levels of the physical, emotional, and psychological burden associated with caregiving (Oechsle et al., 2019). The caregivers’ burden can increase if patients´ needs are not met. The family caregivers’ unmet needs and unresolved problems may harmfully affect their well-being and the patient’s health outcomes (Nelson et al., 2022). Therefore, the path to successful caregiving for cancer patients in palliative care may lie in acknowledging that family caregivers play a fundamental role (Cormio et al., 2014; Pan and Lin, 2022; Rosario-Ramos et al., 2022).

As the delivery of palliative care to cancer patients moves toward community care (Murray et al., 2015), family caregivers are expected to become even more involved in providing palliative care (Finucane et al., 2019). Even the most patient, compassionate and empathetic family caregiver can reach a point where it seems impossible to continue as emotional strains amount (Cormio et al., 2014; Pan and Lin, 2022; Rosario-Ramos et al., 2022). In turn, caregivers’ needs are interconnected with patients’ well-being and will not just decrease their quality of life but negatively impact the patient’s health outcomes (Milbury et al., 2013; Fiest et al., 2018; Leykum et al., 2022).

Addressing burnout of the family caregiver is extremely important (Gérain and Zech, 2019), principally for those providing palliative care. The family caregiver needs to be able to support not just the person at the end-of-life but also the needs of other family members as well as their own.

To address caregiver burnout, interventions must be developed to alleviate it when events occur and prevent it before an event arises (Cochrane et al., 2021; Parola et al., 2022). Healthcare professionals are competent to treat not just the underlying disease but also to provide holistic care to patients, individually and in the context of their families (Cheluvappa and Selvendran, 2022). In this holistic care, healthcare professionals can provide care for nausea or vomiting, pain management, and spiritual and psychological care for patients and their caregivers (Kozlov et al., 2018; Chapman et al., 2022; Coelho et al., 2022). By managing symptoms and providing spiritual and psychological care, healthcare professionals can provide comfort and minimize suffering for the person in palliative care and their family caregiver. For this to happen, it is vital to develop a tailored treatment plan unique to the needs and expectations of the person in end-of-life and his or her family caregiver (Pringle et al., 2015; Birgisdóttir et al., 2021).

Cancer survivors and their family caregivers have specific needs and expectations regarding healthcare professionals’ support. Although several studies explored the cancer survivors’ needs and expectations (Berger et al., 2020; Kim et al., 2020; Johnston et al., 2021; Shamieh et al., 2022), the family caregivers’ perspective is poorly understood. A previous study has identified that patients with advanced cancer and their family caregivers have unmet care needs regarding information, including illness, treatment, and care-related information (Wang et al., 2018). Another study has shown that family caregivers emphasized the need to discuss further treatment plans for their relatives (Chang et al., 2013).

Healthcare professionals are responsible for decisions about how much care patients and family caregivers receive (Haapasalmi et al., 2021). Building knowledge about the cancer patient’s family caregivers’ needs and expectations is essential. This knowledge can enable healthcare professionals to develop an evidenced-base systematic approach to provide optimal care to support patients and their family caregivers. Hence, this study aims to explore the needs and expectations of family caregivers of cancer patients in palliative care.

## 2. Materials and methods

### 2.1. Study design

The phenomena under study are the needs and expectations of family caregivers of cancer patients in palliative care. Given the literature gap, we propose conducting a qualitative descriptive exploratory study.

Descriptive exploratory designs are useful to deepen the knowledge about a particular phenomenon that has not previously been studied in depth in a specific context (Hunter et al., 2019; Doyle et al., 2020).

To ensure the quality of this study protocol, we have followed the consolidated criteria for reporting qualitative research (COREQ) checklist (Tong et al., 2007).

### 2.2. Period

**Figure 1:**
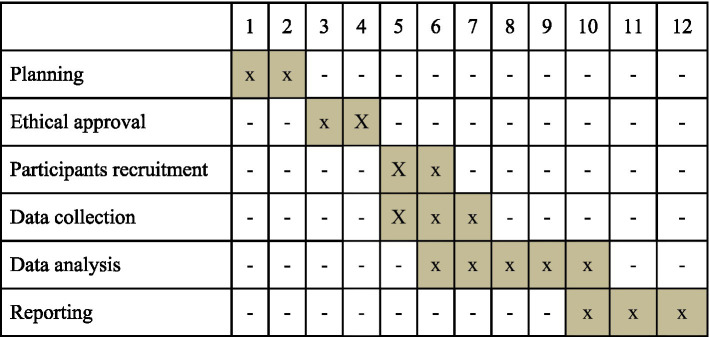
Project schedule.

The study will be developed from October 2022 to July 2023 (Gantt Chart Figure 1).

### 2.3. Population and recruitment

The study will recruit participants (family caregivers) from the palliative care units of two hospital centers in the region of Lisbon and Tagus Valley.

For the purposes of this research, we consider family caregivers as any relative, partner, friend, or neighbor who has a significant personal relationship with, and provides a broad range of assistance for the person receiving palliative care (Family Caregiver Alliance, 2022).

We will publicize the study in the hospitals’ palliative care units *via* a poster placed on the unit’s public information boards. We will also count on the participation of the nursing team to promote the study by word-of-mouth.

**Figure 2:**
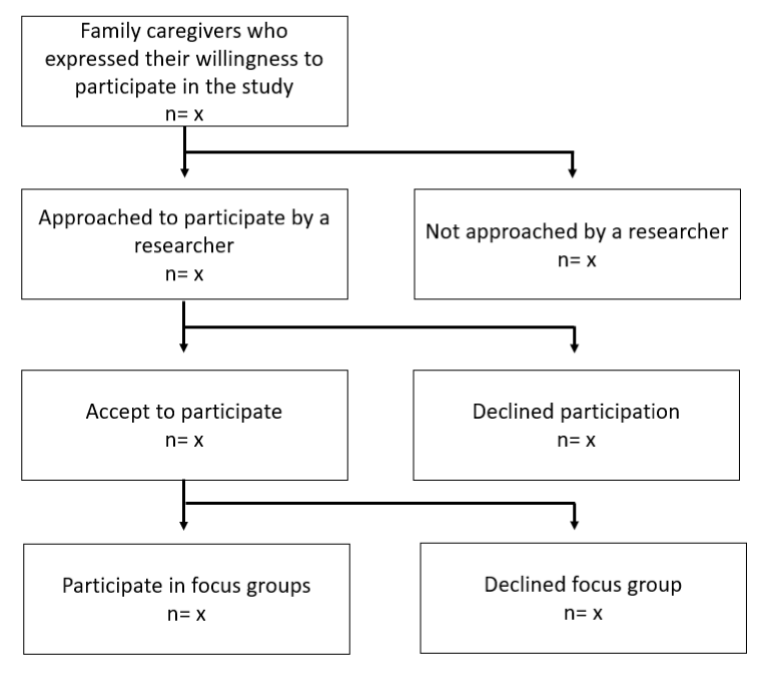
Study participant flow chart.

Figure 2 shows the flow of participants throughout the study.

A purposive sampling approach will be applied to enhance the sample specificity and safeguard that the findings are pertinent to the research background.

This sampling method is widely used in qualitative research because it allows researchers to ensure sample specificity by selecting participants who hold particular characteristics that are pertinent to explore data related to the object of interest (Malterud et al., 2016).

Researchers will purposefully select participants with different ranges of time spent caregiving, time allocated to caregiving (full-time or part-time), and cancer type and stage to include participants that will provide different lived experiences of caring for someone.

To facilitate the purposive sampling, researchers will include in the registration survey questions regarding the number of years in caregiving, time allocated to caregiving and their relatives´ cancer type and stage.

### 2.4. Inclusion criteria

- Be a family caregiver of a cancer patient in palliative care.
- People aged 18 years and above.
- Willingness to participate in the study.
- Ability to understand, and provide informed consent.

### 2.5. Data collection procedures

Data will be collected through focus groups. We choose focus groups because they can generate results quickly, ensuring multiple perspectives. In addition, this research method can offer significant, meaningful insight into participants’ experiences, beliefs, and perspectives (Wong, 2008; Grønkjær et al., 2011; Krueger and Casey, 2014).

All eligible participants who have applied for the recruitment process will be contacted by a member of the research team *via* telephone to present the research project its aims, and verify their willingness to participate and, if so, arrange the practicalities of the focus group discussion. To remind the importance of participation in this research, a telephone call will be made to each participant on the evening of the focus group sessions.

The focus groups will be led by a team of two experienced researchers and skilled moderators who will assume the roles of moderator and note-taker. Both nursing lecturers, one with a Ph.D. in Nursing Sciences (Fernandes et al., 2021, 2022a,b) and the other one with a Ph.D. in Educational Sciences (Ferreira and Nunes, 2019; Ferreira et al., 2022). To increase consistency, the researcher will not switch roles and will not have a prior relationship with the participants.

To ensure discussion generate richly detailed data relevant to the study’s aims, a focus group guide will be structured to foster a natural conversational flow through the discussion and simultaneously explore the particular components of each participant’s unique experience in depth. The guide will consist of a set of open-ended questions aiming to identify and explore the needs and expectations of family caregivers of cancer patients in palliative care. We will use open-ended questions with prompts. For example: Tell me about any needs/burdens/challenges that you experienced as the caregiver. Probes: Please describe the impact on your own health?; Effects on your own esteem?; Impact on your schedule?; Impact on your finances?; Support from other family members?; or Are there any supports that you benefited from? Are there any supports that were not available that you could have benefited from? Probe: Can you tell me more about that?”

The guide will be pilot tested among participants and experts in qualitative research to ensure it is sufficiently explicit, objective, and comprehensive, does not present questions that could be ambiguous or equivocal, and simultaneously allows for retrieval of the required information.

Homogeneity in focus group construction is essential for group interaction and dynamics, but with sufficient variation among participants to allow for contrasting opinions. We will compose the focus groups considering the variables kindship and length of time spent caregiving (Wong, 2008; Grønkjær et al., 2011). A very heterogeneous group can threaten participants and inhibit them from expressing their points of view.

To establish the size of each focus group, we consider that smaller groups (4–6 participants) are preferred when the participants have an intensive experience to share about the topic or when the researcher wants participation from each subject (Wong, 2008; Krueger and Casey, 2014).

The focus group will occur in a private meeting room in the facilities of the League of Friends of the hospital centers. The research team will ensure the chosen location is free of noise in an environment that provides the participant’s privacy and comfort. Only the two moderators and participants will be present during the focus group discussion.

Before beginning the focus group discussion, participants will be asked to fill out a survey to collect their socio-demographic characteristics (e.g., sex, age, marital status, relational status, and time of experience in caring for cancer patients).

Based on the researchers’ previous experience, each focus group is anticipated to last around 90 min.

All focus group discussions will be digitally audio-recorded and transcribed verbatim into a Microsoft Word document. The moderators will make the transcription to ensure the data’s confidentiality and the participants’ anonymity. Later, participants will analyze the transcribed verbatim to consider any discrepancies and provide further clarification that may improve data accuracy.

### 2.6. Data analysis

For the sample characterization, we will use the IBM Statistic Package for the Social Sciences software (IBM Corp. Released 2020. IBM SPSS Statistics for Windows, Version 27.0. Armonk, NY, United States: IBM Corp.) to perform descriptive statistic measures of count, mean, standard deviation, median, minimum, and maximum.

Multiple researchers will analyze and discuss qualitative data as recommended by Corbin and Strauss (2008). The process of data analysis will be executed concurrently with data collection. We will follow the procedures described by Braun et al. (2019) to perform a conventional thematic analysis. For the coding of the transcripts, we will use the coding software QDA Miner Lite database. Two team members will code all focus group discussions to increase the reliability of our coding procedure and minimize personal bias. These researchers will read and listen to the audio transcripts multiple times to allow the results to emerge directly from data analysis rather than from *a priori* expectations or models (Thomas, 2016). This process will facilitate breaking down data into discrete parts, which researchers will closely examine and compare for similarities and discrepancies and later provide codes that, in the final stage, can be grouped into themes and subthemes. Afterward, the two researchers will compare and discuss the broader initial codes, name themes, and subthemes. Any discrepancies will be addressed. A third researcher will analyze the discrepancy if a consensus is not reached.

In the final stage, the two other team members will review the initial data analysis and match each quote to one of the identified themes. Finally, we invite a team of external researchers, experts in qualitative research, to ratify the results.

### 2.7. Data saturation

Since we aim to identify the needs and expectations of family caregivers of cancer patients in palliative care from the perspective of family caregivers, we do not have an estimate of the number of focus groups we will conduct. Rather than setting a fixed sample size, we will focus on the richness of data than on the sample size (Gupta et al., 2018; Vasileiou et al., 2018) and consider saturation, as proposed by Glaser and Strauss (2017). Therefore, focus groups will be conducted until data have reached appropriate consistency to meet the study aims. The criteria to stop data collection is when data regarding a concept reveal no novel properties nor yields any further insights concerning the object of study. Data collection and data analysis procedures will be performed concurrently to allow researchers to modify the focus group guide to incorporate new emerging themes and to stop further recruitment on achieving data saturation (Wray et al., 2007).

### 2.8. Trustworthiness

To guarantee research rigor, we have adopted several procedures. First, the sampling technique will enable researchers to describe the phenomenon under study in all its nuances. Second, we will implement the practices by Nowell et al. (2017) to ensure data trustworthiness. To ensure credibility, the eligible participants will be approached by a team member to establish a good rapport and explain the importance of the study. During focus group discussions, the moderators will ensure that participants will be given enough time to share their feelings and experiences fully. In addition, during data analysis, researchers will discuss and detail every decision until consensus. To guarantee transferability, researchers will incorporate in the final report a detailed description of the study setting and the participants’ characteristics and quotations to allow readers to determine whether a transfer is feasible. Concerning the study’s dependability, every step of the decision-making process will be detailed and documented. Finally, to ensure confirmability, experts in qualitative research external to this research will assess inconsistencies by comparing their perceptions with those of the researchers.

### 2.9. Ethics and procedures

Researchers will conduct the study according to the European Union General Data Protection Regulation and Declaration of Helsinki (as revised in 2013). The Hospital Centers’ Human Research Ethics Committee will review the research protocol. In addition, researchers will ensure that all the participants will have access to an information sheet where it is specified that participation will not affect their relative’s healthcare, their participation is voluntary, and they are free to not reply to some questions, change or review their answers, or withdraw consent at any time.

Written informed consent will be obtained without coercion of study participants before conducting focus groups. Each participant will be assigned a unique code number (for example, P1, P2, P3, etc.) to protect their anonymity and ensure confidentiality.

Only the moderators will have access to the identification sheet. No individual data will be available.

The archive of essential documents will be carried out in a locked file, ensuring their prompt availability, upon request, to the competent authorities. The audio-recorded data will be destroyed after the verbatim transcription. All digital data will be coded and stored on a password-protected computer. All data will remain locked in a file cabinet at Egas Moniz University for 5 years. After this retention period, all data will be destroyed.

## 3. Discussion

Family caregivers play an essential role in the care and support of patients in palliative care and are considered fellow sufferers alongside patients (Cormio et al., 2014; Pan and Lin, 2022; Rosario-Ramos et al., 2022). The literature review showed some studies in this area in other countries (Chang et al., 2013; Wang et al., 2018; Chua et al., 2020). However, in Portugal, no studies were identified, and we believe that legislation on caregivers and cultural aspects can influence needs and expectations. Although researchers have expressed interest in understanding cancer patients’ needs and expectations regarding end-of-life care, the family caregivers’ perceptions are not well known. This research will provide an overview and an understanding of the cancer patient’s family caregivers’ needs and expectations regarding palliative care.

Cancer’s impact on patients and their family caregivers are closely intertwined (Ownsworth et al., 2015; Chua et al., 2020). In addition, as patients reach their end of life, family caregivers may experience an even more significant burden as their family member’s condition deteriorates and physical energy and motions are drained (Chang et al., 2013).

A growing body of literature has conclusively documented that family caregivers support patients but are also affected by the patient’s illness and experience specific needs that often go unmet (Chang et al., 2013; Ullrich et al., 2021). A previous study has highlighted that the family caregivers of older adults have complex needs, and healthcare professionals must provide tailored care to support the transition from family member to caregiver (Ferreira et al., 2020). Cancer survivors in palliative care and their family caregivers have specific psycho-social and educational needs and face several barriers to receiving high-quality, patient-and-family-centered care (Moore et al., 2021; Nelson et al., 2022). Optimal care is needed to address this problem. Inconsistent care mismatched with patients’ and caregivers’ needs can lead to poor health outcomes and increased healthcare expenditure (Wang et al., 2018).

The needs and expectations of family caregivers should be comprehensively assessed for palliative care services to provide patient-and-family-centered care. Since patients with cancer at the end of life suffer from several physical and psychological symptoms, it is understandable that we identify more needs in this study.

We draw attention to the fact that this study can have significant implications from both clinical and research perspectives. We expect that the findings from this study contribute to healthcare professionals providing an evidence-based approach to delivering patient and family-centric palliative care. Furthermore, it has the potential to identify gaps in care and input feedback in the development of interventions, such as the production of additional information materials (Moore et al., 2021) and support programs and services to address the critical areas of identified needs of family caregivers. Additionally, it can allow stakeholders to take the initiative to develop a comprehensive support system for cancer patients in palliative care and their family caregivers. Therefore, the findings of this study can have practical implications not only for family caregivers and healthcare professionals but also for healthcare management and policymakers.

Similar to previously published study protocols (Apadula et al., 2022; Chen et al., 2022; Laker et al., 2022), with the development of this protocol, we seek to present the aims, methodological approach and plan to operationalize the research.

There are several possible limitations in the study that will require the development of strategies to minimize their occurrence. First, using focus groups as the data outcome might be considered a limitation. However, we believe that this data set can be regarded as best suited to answer the aims of this study and address the gap in the literature regarding the needs and expectations of family caregivers of cancer patients in palliative care. Second, a focus group depends deeply on a discussion and may limit participants in producing helpful discourse. This research method can also inhibit participants from revealing sensitive topics. In addition, the contributions of each participant may be disproportionate if there is an outspoken group member, and the participants may influence each other, which can affect answers. To overcome this limitation, we rely on the experience of the leading researcher to facilitate the discussion and help produce helpful information. Third, researchers are aware that the usefulness of the findings and resulting recommendations are directly linked to input, that is, the family caregivers’ perspectives regarding the object of study. Therefore, we aim to explore different perspectives so that the data will have the potential to inform policies and practices about supporting family caregivers to minimize caregiver burden. Fourth, researchers recognize that the participants’ perceptions can diverge from what they report due to a lack of confidence in the researchers’ ability to safeguard anonymity or protect their identity, values, or beliefs. Therefore, the researchers have adopted several procedures, detailed throughout this report, to ensure research rigor and compliance with the ethics procedures.

## 4. Conclusion

It is vital to understand the needs and expectations of family caregivers of cancer patients as a means to develop and improve care practices. This study will contribute to the limited literature regarding the needs and expectations of family caregivers of cancer patients in palliative care. It will identify gaps in care and provide data that can support healthcare professionals in delivering evidence-based care to family caregivers. In addition, it can generate data that support stakeholders in developing a comprehensive support system for both cancer survivors in palliative care and their family caregivers.

## Author contributions

JF, JD, CC, AS, SF, AA, CBe, CBa, RF, and CG: conceptualization. JF, JD, CC, AS, SF, AA, CBe, LM, SR, RF, CBa, MS, and CG: methodology, writing—original draft preparation, and writing—review and editing. JF and CG: supervision and project administration. All authors contributed to the article and approved the submitted version.

## Funding

This research received no external funding.

## Conflict of interest

The authors declare that the research was conducted without any commercial or financial relationships that could be construed as a potential conflict of interest.

## Publisher’s note

All claims expressed in this article are solely those of the authors and do not necessarily represent those of their affiliated organizations, or those of the publisher, the editors and the reviewers. Any product that may be evaluated in this article, or claim that may be made by its manufacturer, is not guaranteed or endorsed by the publisher.
